# Learning Bayesian Networks from Correlated Data

**DOI:** 10.1038/srep25156

**Published:** 2016-05-05

**Authors:** Harold Bae, Stefano Monti, Monty Montano, Martin H. Steinberg, Thomas T. Perls, Paola Sebastiani

**Affiliations:** 1 Oregon State University, College of Public Health and Human Sciences, Corvallis, 97331, USA; 2 Boston University, Department of Medicine, Boston, 02118, USA; 3 Harvard Medical School, Department of Medicine, Boston, 02115, USA; 4 Boston University, Department of Biostatistics, Boston, 02118, USA

## Abstract

Bayesian networks are probabilistic models that represent complex distributions in a modular way and have become very popular in many fields. There are many methods to build Bayesian networks from a random sample of independent and identically distributed observations. However, many observational studies are designed using some form of clustered sampling that introduces correlations between observations within the same cluster and ignoring this correlation typically inflates the rate of false positive associations. We describe a novel parameterization of Bayesian networks that uses random effects to model the correlation within sample units and can be used for structure and parameter learning from correlated data without inflating the Type I error rate. We compare different learning metrics using simulations and illustrate the method in two real examples: an analysis of genetic and non-genetic factors associated with human longevity from a family-based study, and an example of risk factors for complications of sickle cell anemia from a longitudinal study with repeated measures.

Bayesian Networks (BN) are flexible probabilistic models that have become increasingly popular in many fields, including genetics and genomics^1^,^2^,^3^,^4^,^5^. There are well established approaches to structure and parameter learning of a BN from a random sample of independent and identically distributed (IID) observations^6^,^7^. However, many observational studies are designed using some form of clustered sampling that introduces correlations between the observations within the same cluster^8^. Examples of such designs includes family-based studies, in which families represent clusters and relatives within the same family cannot be assumed independent because they share more genetic and non-genetic factors than unrelated individuals, and longitudinal studies with repeated measurements of the same individuals over time^9^. It is well known that ignoring the correlation between observations can impact the false positive rates of regression methods^10^, and the same problem is likely to persist with using BNs. As an example, Fig. 1 illustrates the effect of ignoring the correlation between observations when learning the network structure using three common model selection metrics. Regardless of the model selection metrics, both the false positive rates and family-wise error rates are greatly inflated when the correlation is ignored.

Linear mixed models and generalized linear mixed modeling are two popular approaches to address the issue of correlated data^11^,^12^, but they are not directly applicable to BN modeling. In this paper, we propose a parameterization that extends mixed effects regression models to BNs and can be used for both structure and parameter learning from correlated data. The parameterization can work with a mix of variable types including categorical, continuous, and time-to-event data. In the next section we briefly review methods for learning BNs from independent and identically distributed observations, and describe mixed-effects regression models for the analysis of correlated data. We next extend mixed-effects regression models to BNs and then present the results of simulation studies that describe the inflation to the Type I error due to ignoring correlated data and compare different model selection metrics that can be used for learning mixed-effects BNs. We illustrate our proposed approach in two real data examples. Finally, conclusions and suggestions for further work are provided.

## Background

### Learning Bayesian Networks from Independent and Identically Distributed Observations

A BN is a vector of random variables *Y* = (*Y*_1_, …, *Y*_*v*_) with a joint probability distribution that factorizes according to the local and global Markov properties represented by the associated directed acyclic graph (DAG)^13^,^14^,^15^. The *local Markov property* states that a variable is independent of its non-descendants given its parents, where the non-descendant of a variable *Y*_*i*_ are all variables linked to *Y*_*i*_ through a directed path pointing to *Y*_*i*_. The *global Markov property* states that a variable is independent of all the remaining variables in the graph conditionally on its Markov blanket that is defined by the parent nodes, children nodes and additional parents of the children nodes.

There are well established approaches to structure learning of BNs^6^,^7^,^13^ that use either exact Bayesian criteria based on the marginal likelihood 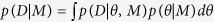, or asymptotic criteria such as 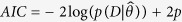, or 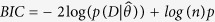 where *D* denotes the sample of size *n*, *M* denotes the BN structure, *θ* is a vector of *p* model parameters, *p*(*D*|*θ*, *M*) and *p*(*θ*|*M*) denote the likelihood function and the prior distribution of the parameters, and 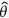 is the maximum likelihood estimate of *θ*. Popular approaches such as the *K2* algorithm^16^ leverage the decomposability of the likelihood function to break down the model search into a modular search of the dependency of each node on the parent nodes^17^,^18^. The decomposability of the likelihood is based on the factorization of the probability distribution of the variables *Y* = (*Y*_1_, *Y*_2_, …, *Y*_*v*_) according to the local Markov property described by a DAG *M*


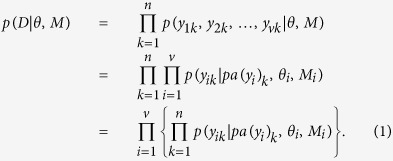


**Figure 2:**
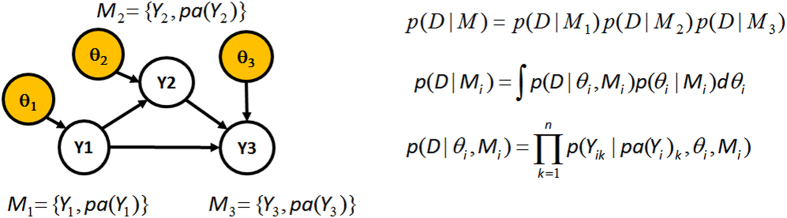
Example of BN with 3 observable variables (*Y*_1_, *Y*_2_, *Y*_3_) and parameter vectors *θ* = (*θ*_1_, *θ*_2_, *θ*_3_). If there are no missing data, the observations are independent, and the prior distribution of the parameters follow Hyper-Markov law, then the marginal likelihood *p*(*D*|*M*) factorizes into a product of 3 local marginal likelihood functions.

In Equation (1), *pa*(*y*_*i*_) denotes the observable parents of the variable *Y*_*i*_ in the model *M*, while *y*_*ik*_ and *pa*(*y*_*i*_)_*k*_ denote the observed value of *Y*_*i*_ and its parent nodes in the k-th sample unit. Each sub-model *M*_*i*_ specifies the set of parents of the variable *Y*_*i*_ (see Fig. 2), so that *M* = (*M*_1_, *M*_2_, …, *M*_*v*_). We denote by *y*_*k*_ = (*y*_1*k*_, *y*_2*k*_, …, *y*_*vk*_) the vector of values of the variables measured in the *k*-th sample unit, and by *θ* the set of parameters *θ* = (*θ*_1_, …, *θ*_*v*_), where each *θ*_*i*_ can itself be a vector of parameters indexing the conditional distribution of the variable *Y*_*i*_ given its parents *pa*(*y*_*i*_).

In addition to the local Markov property, efficient Bayesian computations rely on a factorization of the prior distribution for the vector of parameters *θ*. Dawid and Lauritzen^19^ described general Hyper-Markov laws that assume certain marginal and conditional independences of the parameters to produce this factorization:


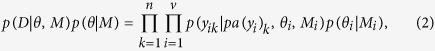


that is used to compute the marginal likelihood as the product:


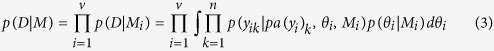


that can be used for a modular Bayesian model search. All of these proposed approximations assume that the observations are independent or exchangeable.

### Mixed-Effects Regression Models

Mixed effects regression modelling has emerged as one of the most popular method to analyze correlated data^11^. Let *Y* denote the observations of *n* subjects from *m* clusters, and suppose that *Y* follows a multivariate normal distribution. A linear mixed effects model for *Y* is described by the equation:


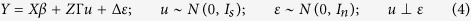


in which *X* is an *n* × *p* matrix of regression coefficients for the fixed effects *β*, *Z* is an *n* × *s* matrix of known coefficients, Γ and Δ are *s* × *s* and *n* × *n* matrices of parameters that describe the correlations between observations, *u* is a vector of *s* × 1 random effects, and *ε* is a vector of *n* × 1 error terms^11^,^20^. The model specifies that


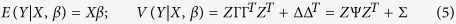


so that the correlation between the observations is described by the matrices Ψ and Σ. If both Ψ and Σ are block diagonal matrices, the parameterization is the independent cluster model, in which subjects from *m* different clusters are independent, but they are correlated within the same cluster. Note that the parameterization in Equation (4) assumes that the vectors *u* and *ε* are standardized to have variances equal to 1 and they are independent, while the variance components of *Y* are the elements of the matrices ΓΓ^*T*^ and ΔΔ^*T*^. For analysis of time-to-events data with proportional hazard models, the random effects are usually modeled in the log-hazard function or using a frailty term with gamma distribution^21^,^22^,^23^,^24^. For categorical data modeled within the framework of generalized linear models, the random effects are modeled on the scale of the linear predictors^25^. These models make the additional assumption that the observations are independent, conditionally on the random effects.

**Figure 3:**
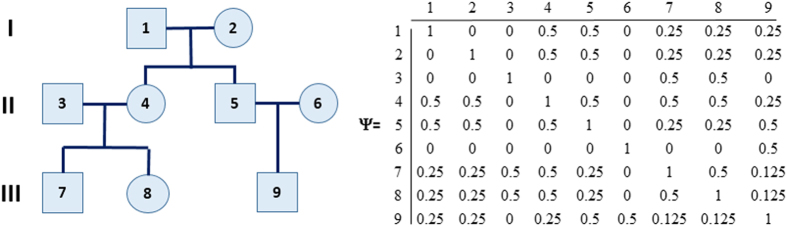
An Example Pedigree and Corresponding Additive Genetic Relationship Matrix. The kinship matrices contain pairwise kinship coefficients between pairs of family members and these coefficients represent the probability that two individuals share the same gene allele by identity by descent. The covariance between two family members with kinship coefficient *k*_*ij*_ is 2*k*_*ij*_*γ*^2^ where *γ*^2^ represents the genetic variance.

Non-Bayesian inference on the fixed effects parameters typically uses the marginal approach based on the *integrated likelihood*:


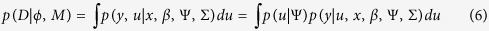


where *ϕ* represents the vector of fixed effects *β* and variance parameters in Ψ and Σ. The integrated likelihood can be computed in closed form when errors and random effects are normally distributed, but numerical approximations are needed for non-linear/non-normal models^26^. The integrated likelihood is used to find maximum-likelihood estimates of the fixed effects and variance components. Common parameterizations of the random effects include exchangeable correlation, in which within-cluster pairwise correlation is assumed constant, and auto-regressive correlation. When clusters are families, the correlation between family members depends on the degree of relatedness and kinship coefficients that represent the probability of alleles transmitted identically by descent between pairs of family relatives^27^ (See Fig. 3).

Several model selection criteria have been proposed for selection of fixed effects in mixed effects^11^, including *AIC* and *BIC* that can be computed using the integrated likelihood but there is no consensus on the appropriate correction parameters. Specifically, it has been argued that the overall sample size may not be the correct quantity to use when the data are correlated and the effective number of parameters may be unclear in models that includes several random effects^28^,^29^. Modified versions of *BIC* that have been proposed, say 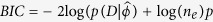 where *n*_*e*_ is an estimate of the *effective sample size*, use a reduced sample size to account for the correlation between observations^11^. We will use these three proposed corrections of the sample size in the simulation study:

#### Jones’ correction

*n*_*e*_ = 1^*T*^*C*^−1^1, where 1 is the unit vector, and *C* is the correlation matrix that can be estimated from the covariance matrix *V* = *V*(*Y*|*X*, *β*) = *Z*Ψ*Z*^*T*^ + Σ^30^.

#### Yang’ correction


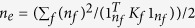
 applies to family based data. It assumes that the data are from families of size *n*_*f*_ and each *K*_*f*_ denotes the kinship matrix for the *f*th family^31^.

#### Liberal correction

*n*_*e*_ = *n*_*c*_ where *n*_*c*_ is the number of clusters. This is the most liberal correction, in which a cluster represents a single sample unit.

### Mixed-Effects Bayesian Networks

We propose a mixed-effects type parameterization of a BN that can be used for structure and parameter learning from correlated observations. The rationale of our approach rests on the observation that in the mixed effect regression model in Equation (4) we introduce and model the correlation between the sample units through the vector of random effects *Z*Γ*u*, with variance-covariance matrix *Z*Ψ*Z*^*T*^, where Ψ = ΓΓ^*T*^.

To extend this idea to a BN with variables *Y*_1_, …, *Y*_*v*_, consider first this simple situation. Suppose *v* = 2 and let *Y*_1_ be parent of *Y*_2_, with *E*(*Y*_2*k*_|*Y*_1*k*_) = *β*_1_*Y*_1*k*_ and (*Y*_1_, *Y*_2_) follows a bivariate normal distribution. To estimate the coefficient *β*_1_ from a sample of observations that are not independent and have correlation structure known up to some parameters, one can use the mixed effect regression model


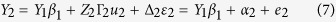


where *e*_2_ represents the iid sampling error for the variable *Y*_2_ and we assume 
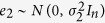
, and *α*_2_ is a vector of random effects distributed as 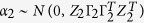. With this parameterization, 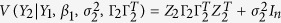, if *α*_2_ and *e*_2_ are independent, and the likelihood function used to estimate the parameter *β*_1_ takes into account the correlation of the observation through the matrix 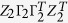. The examples at the end of this section show how to specify 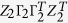 in two common situations of family-based studies and longitudinal studies with repeated measures. We can extend this parameterization to all variables in a network but some assumptions on the relation between random effects are needed to maintain the decomposability of the likelihood function.

**Figure 4:**
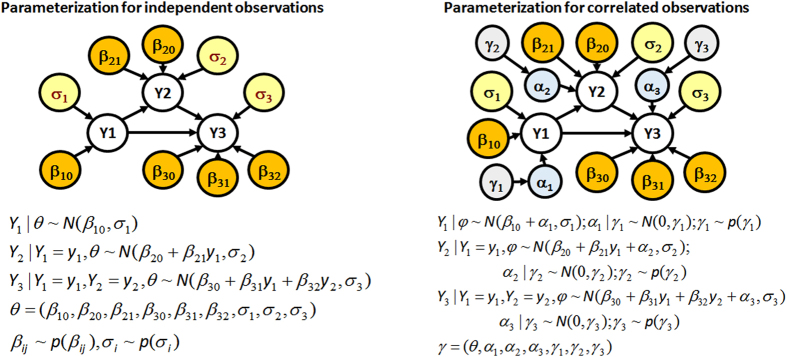
Left panel: common parameterization of a simple directed graphical model with 3 observable, Gaussian variables (*Y*_1_, *Y*_2_, *Y*_3_), conditional of the parameter vector *θ*. Nodes in orange are the parameters that define the conditional parent-children distribution of the observable variables (fixed effects), while the nodes in yellow are nuisance parameters. Right panel: our proposed parameterization when both the dependency structure and conditional probability distributions need to be estimated from correlated data. The random effects *α* (blue nodes) have probability distributions that depend on parameters *γ* (lavender nodes). Both parameters *γ* and random effects *α* are used to model the correlation between observations as in Equation (4).

We proceed by introducing a set of random effects *α* = (*α*_1_, …, *α*_*v*_), in which each *α*_*i*_ is a *n* × 1 vector of correlated random effects associated with *Y*_*i*_. The random effects can be interpreted as additional parameters that augment the parent set of each variable *Y*_*i*_ as (*pa*(*Y*_*i*_), *θ*_*i*_, *α*_*i*_, *γ*_*i*_) where the vector *θ*_*i*_ represents the parameters of the conditional distribution of each node *Y*_*i*_|*pa*(*Y*_*i*_) that we would ordinarily use if observations were IID, and the vector *γ*_*i*_ represents the variance parameters of *α*_*i*_ that model the correlation between observations. We assume that the distribution of the random effects *α*_*i*_ depends only on the variance parameters *γ*_*i*_, so that *θ*_*i*_ and *α*_*i*_ are independent given *γ*_*i*_. For example, if the variables *Y*_*i*_ follow normal distributions and the parent-children relation are described by regression models, the parameter vector *θ*_*i*_ includes the regression coefficients *β* and the uncorrelated error variance terms, while *γ*_*i*_ represents the correlation parameters. See Fig. 4 for an example.

Let *ϕ* denote the overall set of parameters (*θ*, *α*, *γ*) of the joint probability distribution of the variables *Y*_1_, …, *Y*_*v*_ and we assume *global independence of the parameters*


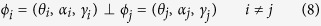


so that we can write the product of the global likelihood function and parameter prior distribution for data *D* as:


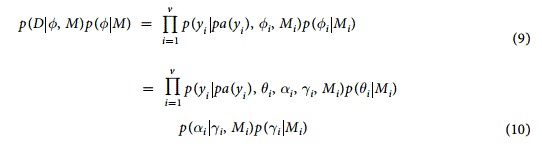


This factorization of the augmented likelihood can be used for local computations using information based criteria or other Bayesian criteria. For example, conditionally on *θ* = (*θ*_1_, …, *θ*_*v*_) and *γ* = (*γ*_1_, …, *γ*_*v*_), the integrated likelihood can be computed as:


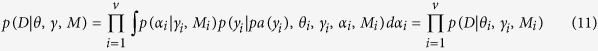


where *p*(*D*|*θ*_*i*_, *γ*_*i*_, *M*_*i*_) can be computed exactly for normally distributed variables (see^11^) or using numerical approximations in other cases as shown in^26^. Maintaining the product-form of the likelihood has the benefit that the search of the best dependency structure among the variables *Y*_1_, …, *Y*_*v*_ can be conducted in a modular way, by finding the optimal set of parents of each variable *Y*_*i*_ that optimizes either the marginal likelihood, or the marginal *BIC* or *AIC* based on integrated likelihood.

The variables *Y*_*i*_ can follow a normal distribution, or non-normal distributions such as a Poisson distribution or multinomial distribution for categorical data, or survival distribution for time-to-event data. In the non-normal data case, random effects can be included in the log-transformed parameterization of the mean (Poisson/multinomial data), or the log-hazard function (time-to-event data). Once the best dependency structure is selected, the conditional distributions of each local model can be estimated using MCMC methods, or large sample approximations.

### Example of Family-Based Data

Suppose that study subjects are clustered into *m* families with different familial relations, then the within-family correlation of a variable *Y* can be described by the random effect *α*|*γ*^2^ ~ *N*(0, 2*γ*^2^*K*), where *γ*^2^ is the genetic variance to be estimated from data, and the matrix *K* can be derived from the kinship coefficients as in Fig. 3. By using the singular value decomposition of 2*K* = *USU*^*T*^, with *UU*^*T*^ = *U*^*T*^*U* = *I*_*n*_, we can define Γ = *γS*^1/2^, *Z* = *U* and *u* ~ *N*(0, *I*_*n*_), so that *α* = *Z*Γ*u* = *γUS*^1/2^*u* and the variance covariance matrix of *α* is *γ*^2^*US*^1/2^*S*^1/2^*U*^*T*^ = 2*γ*^2^*K*. To extend this parameterization to a Bayesian network with *Y*_1_, …, *Y*_*v*_ variables, for each *Y*_*i*_ we can then define *α*_*i*_ = *γ*_*i*_*US*^1/2^*u*_*i*_, with *u*_*i*_ ~ *N*(0, *I*_*n*_). With this parameterization, we allow the genetic variance 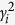 to vary for each variable *Y*_*i*_, but the matrices *U* and *S* will be the same for each *Y*_*i*_, as the kinship matrix *K* is study specific. We can assume a Gamma prior on each parameter 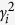, and independence of *θ*_*i*_ and 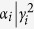 for each *i* = 1, …., *v*, and independence of *α*_*i*_, 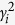 and *α*_*j*_, 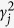 for all *i* ≠ *j* to derive the above factorization of the likelihood.

### Example of Repeated Measures

Suppose that data are from a longitudinal study with repeated measures per subject, and we stack the repeated measures per subject, so that the overall size of the data set *D* is 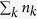 where *n*_*k*_ denotes the number of repeated measures of the *k*th subject. Clearly, the repeated measures of each individual are correlated and the within subject correlation in each variable *Y*_*i*_ can be described by a vector of random effects *α*_*i*_|*γ*_*i*_ ~ *N*(0, *γ*_*i*_Ψ_*i*_) where the matrix Ψ_*i*_ is a block diagonal matrix with blocks that can be parameterized using exchangeable correlation, or autoregressive structure.

## Simulation Studies

We conducted simulation studies to examine the effect of ignoring the correlation between observations in structure learning of a BN. We also compared false positive rate and power of the modifications of *BIC* and *AIC* for learning a BN from correlated data. For simplicity, we focused on the forward search procedure of the *K*2 algorithm^16^. We considered two scenarios: continuous data that follow normal distributions, and time-to-event data modelled using Cox proportional hazard regression. In the first case, we used the closed form solution to the integrated likelihood that allows for efficient computations of likelihood based model selection criteria^11^. In the second case, we used the numerical approximation of the marginal likelihood that can be derived assuming normally distributed random effects in the log-hazard scale^22^,^32^. In both simulations we generated data assuming that data are from a family based study design.

### Continuous Data

We generated correlated observations borrowing the family structure from the Long Life Family Study (LLFS): a study of healthy aging that enrolled individuals from families with longevity and healthy aging in the United States and Denmark between 2006 and 2009^33^,^34^. A typical family structure in the LLFS has a proband and consenting siblings, their offspring and spouses. For this simulation study, the total sample size was 4656 and the number of families was 582. With a kinship matrix from each family *K*_*f*_, the variance-covariance matrix of the observations is the 4656 × 4656 matrix:


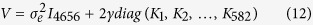


where 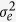 is the error variance, and *γ* is the “genetic variance”. To simulate normally distributed data with this variance-covariance matrix, we fixed the error variance 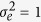 and varied the genetic variance to be *γ*^2^ = 1/3, 1, 3 to simulate genetic traits with heritability 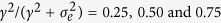, representing the situations of 25%, 50%, and 75% of the trait variability due to genetics and the rest to other non-genetic factors. To generate correlated data, in each simulation a vector *Z* of independent and normally distributed observations was generated and transformed into *Y* = *UD*^1/2^*Z* where *U* and *D* are the matrix of eigenvectors and eigenvalues from the spectral decomposition of the variance-covariance matrix *V*. This transformation guarantees that *V*(*Y*) = *UD*^1/2^*V*(*Z*)*D*^1/2^*U*^*T*^ = *V*. In each run, we also included 10 null covariates. In this simulation study, the null covariates were common single nucleotide polymorphisms (SNP) with minor allele frequency >5%, which were randomly selected from the real genome-wide genotype data from LLFS. Each simulated data was analyzed using a forward search with *BIC* and *AIC* and the *LRT* at *α* = 0.05 ignoring the correlation in the data. The data were also analyzed using *BIC*, *AIC* and the *LRT* based on the intergrated likelihood, 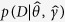 to account for the correlation in the data. Four variants of *BIC* were used based on different effective sample sizes: *n*_*e*_ = 4656 (full sample size); *n*_*e*_ = 2796 (Jones’ correction); *n*_*e*_ = 1768 (Yang’s correction); and *n*_*e*_ = 582 (most conservative sample size). The simulation was repeated 1,000 times.

Table 1 shows the number of false positive covariates that were selected with the forward search using the 9 criteria, the overall number of tests conducted during the forward search, false positive rate (probability of Type 1 error in one test: *BIC*, *AIC* or *LRT*) and family wise error rate (probability of one or more errors in the overall search) when the heritability is 0.5. The false positive rate was calculated by dividing the sum of all false positive covariates by the total number of tests. The full set of results for different heritability estimates can be found in the Supplementary Materials. The results show an inflation of both error rates when the correlation in the data is ignored, with a 55% increase of the family wise error rate for the *LRT*, and a 267% increase for the *BIC*. The inflated Type I error will repeat for each search of parent-child dependency in the network and result in highly connected networks. The false positive rate of the *LRT* based on the integrated likelihood that accounts for the correlation in the data is slightly below the nominal level (0.0432). The various corrections of the *BIC* result in small false positive and family wise error rates. Using the full sample size as the effective sample size in the *BIC* is an over-correction that results in a very conservative scoring metrics. Decreasing the effective sample size makes the *BIC* score more liberal with a modest increase of both false positive and family wise error rates. Although these small error rates of BIC seem desirable, the question is their effect on the true positive rates of the different scoring metrics.

We compared the power of different variants of *BIC* to the power of the *LRT*_*M*_ using the significance threshold determined from the false positive rates in Table 1. To do so, we ran 3 additional simulations in which the variable *Y* was generated from a multivariate normal distribution with variance-covariance structure as described above. In these scenarios, we modelled the expected value of the variable *Y* as a linear function of 3 true covariates that were also generated from a multivariate normal distribution with different amount of correlations. Three sets of regression parameters were chosen to represent the situations of weak, moderate and strong covariate effects such that the first scenario included 3 weak effect covariates, the second scenario included 3 moderate effect covariates, and the third scenario included 3 strong effect covariates. Power was defined as the probability of detecting all three true covariates in each run. The results are summarized in Table 2 and show that the *LRT*_*M*_ has consistently higher power than the BIC for all different corrections, when the false positive rates are kept equal. For instance, in the presence of covariates with moderate effects, the *BIC*_*M*_ detects the 3 covariates 29.5% of the time, whereas the *LRT*_*M*_ detects the covariates 31.4% of the time, which is an increase in power by 1.9%. The most liberal correction of the BIC, with effective sample size equal to the number of clusters, appears to provide a reasonable compromise, and is essentially equivalent to using the LRT.

### Time-to-event Data

To simulate time-to-event data, we again borrowed the family structure from the LLFS and modified the simulation scheme from^35^ by inducing correlation with log-normal frailty (random effects). The baseline survival time was simulated from a *Weibull* (2, 2). We simulated the correlated trait such that:


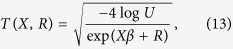


where


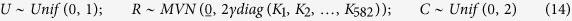


so that the event time is defined as *t* = *min*(*T*, *C*) and the censoring indicator is *δ* = *I*(*T* ≤ *C*). The correlation among observations are induced by the inclusion of random effects term *R* on the log-hazard scale. The rest of the simulation scheme was very similar to the case of continuous data, except for *BIC*, where the effective sample size was the number of events as suggested by^36^.

Table 3 shows the number of false positive covariates that were selected with the forward search using the 6 criteria, the overall number of tests conducted during the forward search, and both the false positive rate and the family wise error rate when the heritability is 50% on the log-hazard scale. The full set of results for different heritability estimates can be found in the Supplementary Materials. The results show an inflation of both error rates when the correlation in the data is ignored, with a 25% increase of the family wise error rate for the *LRT*, and a 56% increase for the *BIC*. The false positive rate of the *LRT* based on the integrated likelihood is slightly below the nominal level (0.0470), while the traditional *LRT* exhibits inflated Type 1 error rate of 0.0629. Consistent with the results from the continuous data, *AIC* is the most liberal metric.

We compared the power of *BIC*_*M*_ based on integrated likelihood to the power obtained from the *LRT*_*M*_ using the significance threshold determined from the false positive rates in Table 3. Again, we ran 3 additional simulations in which the correlated survival trait was generated as described above with sets of 3 true covariates of different strengths. The results summarized in Table 4 show that the *LRT*_*M*_ has consistently higher power in all cases regardless of the heritability estimates, which is consistent with results from the continuous data. For instance, in the presence of covariates with moderate effects when *h*^2^ = 0.50, *BIC*_*M*_ detects the 3 covariates 25.5% of the time, whereas the corresponding *LRT*_*M*_ detects the covariates 28.5% of the time, which is an increase by 3.0%.

These results emphasize the need to account for correlation in the data to avoid an unnecessary inflation of the false positive error rates. Moreover, after controlling for the Type 1 error, the *LRT*_*M*_ appears to have comparable power to the *BIC* based on integrated likelihood in both cases of continuous and time-to-event data. In practical application, using the *LRT* is an appealing approximate solution that avoids the problem of choosing the appropriate number of parameters, and also control well the Type 1 error.

## Application

We applied the proposed approach in the two real data and compared to the BNs constructed when ignoring correlations in the data.

In the first example, we built a BN to examine the associations between genetic data, blood biomarkers, socio-demographic factors, and life span using data from the LLFS. The genetic variants were unlinked SNPs in the 23 genes of the insulin and insulin-like growth factor 1 signaling (IIS) pathway that were found associated with age at death using single SNP analysis (*i.e.* testing the association one SNP at a time using Cox proportional hazard regression adjusted for family structure with a significance threshold of 0.005). This pathway is considered as one of the most important pathways in aging^37^. Table 5 summarizes the gene, chromosome, and the number of tested SNPs per gene. There was a total of 13 common SNPs that were individually associated with age at death, adjusting for sex. In a joint model that included these 13 SNPs as covariates, 6 of them were still associated with age at death at the *p*-value threshold of 0.005. Given the large number of tested SNPs, the *p*-value threshold of 0.005 may appear too liberal but the goal of this preliminary analysis was primarily to obtain a candidate list of genetic variants to be considered in building the BN.

The question we were trying to answer with the BN was whether some of these direct associations between SNPs and lifespan could be explained through associations with blood biomarkers such as serum levels of DHEA (a steroid hormone linked to muscle loss in aging), insulin growth factor 1 (IGF-1), transferrin receptors (Tr), and hemoglobin (Hgb). All these biomarkers are related to aging and would provide targets to develop intervention for healthy aging^38^. Additional variables in the network were age at enrollment (Age.E) and follow-up survival time (FUS) censored at last contact for living subjects. We also included an indicator variable (Birth Year Cohort: BYC) that accounted for possible secular trend, and sex. To build the BN, we used the search procedure of the *K*2 algorithm, and we considered all possible orderings of the other variables with the exception of the SNPs that, for biological reasons, were considered as root nodes in the BN. The follow-up survival time was considered as possible child of all the other nodes. For each possible ordering of the variables, a BN was built by fitting appropriate mixed effects regression models of follow-up survival time (using mixed effect Cox proportional hazard regression), age at enrollment, the four biomarkers (using linear mixed model), and by identifying statistically significant predictors through a forward search. Based on the simulation study, the likelihood ratio test from mixed effects model was used for model selection criteria by applying a Bonferroni correction at each node.

**Figure 5:**
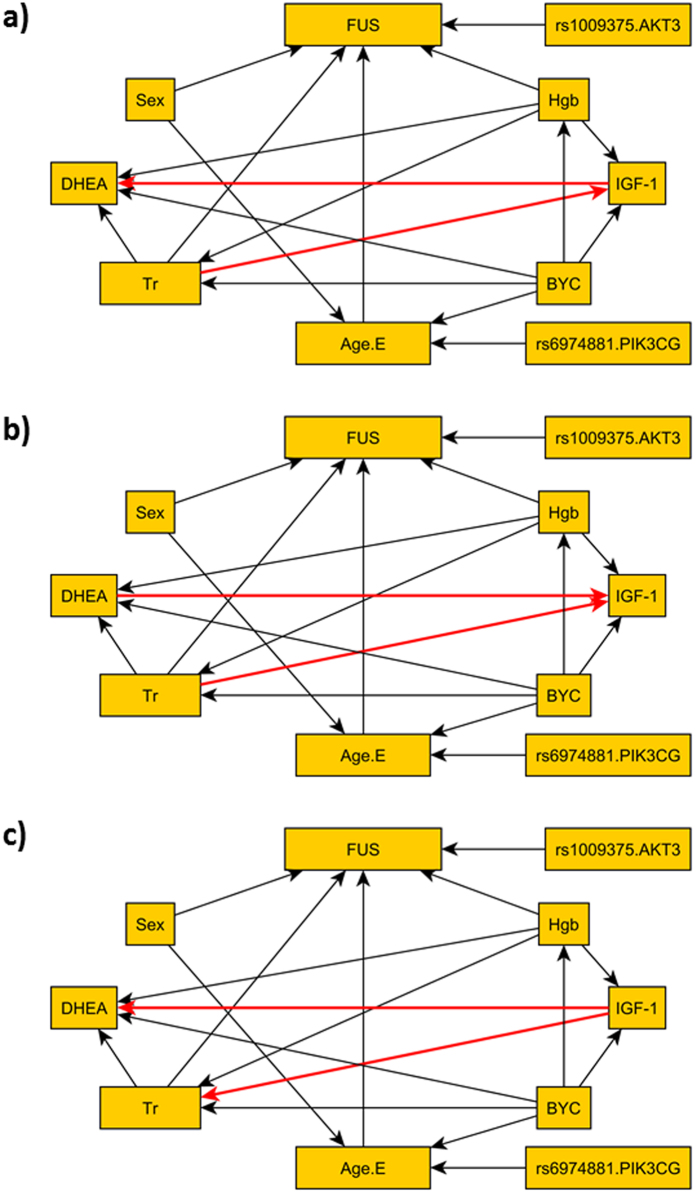
Top 3 BNs built using the proposed parameterization that dissect the associations of SNPs in genes of the IIS pathway through effects on blood biomarkers. The different edges among the three networks are colored in red.

The three BNs with largest global likelihood are depicted in Fig. 5 and have very similar structures, with directions of few edges switched (edges colored in red in the figure), and the Markov Blankets (MB) of the variables in these 3 BNs in Table 6 areidentical. Only two SNPs remained in the model, one (rs1009375, in the proximity of AKT3, linked to glicemic control) is directly associated with follow-up survival and another (rs6974881, in PIK3CG, linked to inflammation) is directly associated with age at enrollment. The results suggest that genetic variants in the IIS pathway do not affect age at death through these 4 biomarkers.

**Figure 6:**
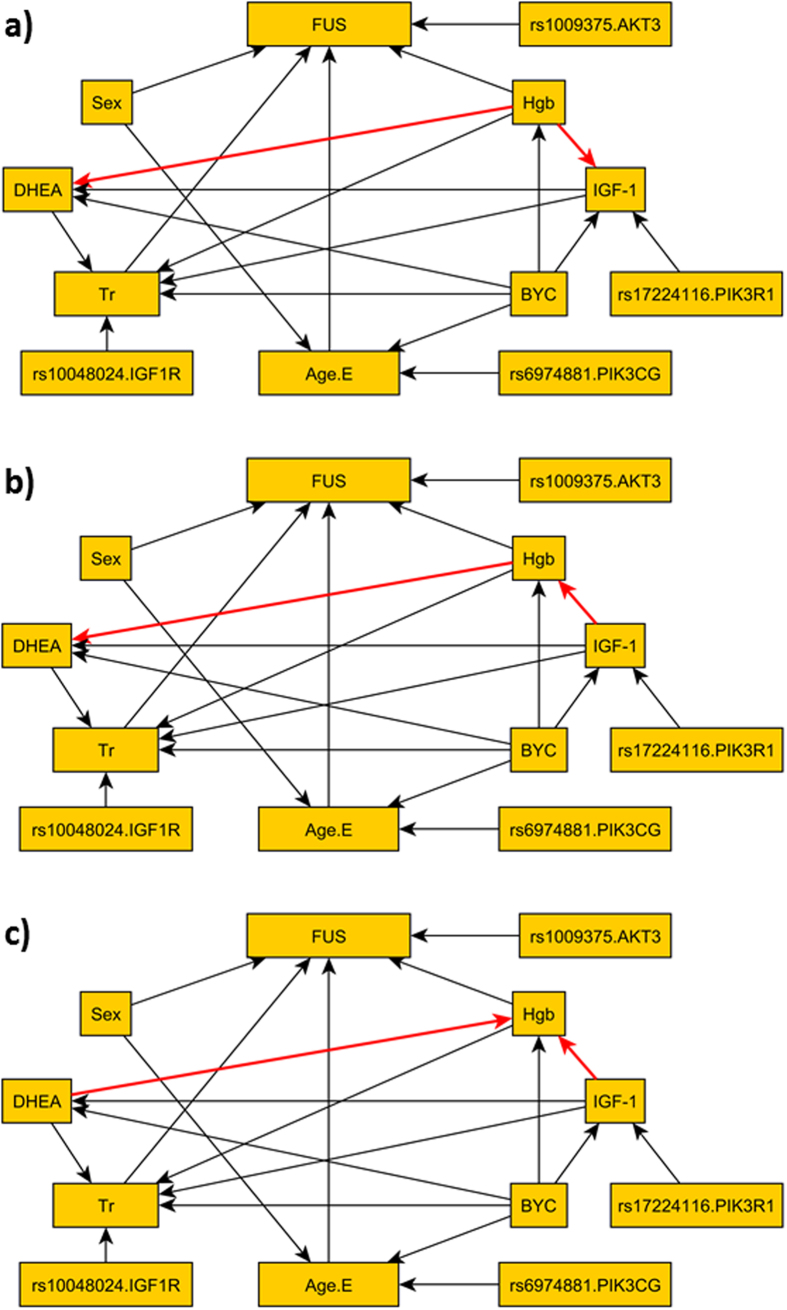
Top 3 BNs built ignoring the familiar correlations in the data used in Fig. 5. The different edges among the three networks are colored in red. Compared to the BNs in Fig. 5, two additional SNPs rs17224116 and rs10048024 are added to the models.

We also built the BN ignoring the familiar correlations in the LLFS data using the *LRT*, and the three BNs with largest likelihood are depicted in Fig. 6. The overall structures are very similar to the top three BNs built under the proposed parameterization. However, in each of these BNs, two additional SNPs (rs17224116 and rs10048024) show significant dependency with transferin receptor level (node Tr) and IGF-1 levels. Based on the results of the simulations that showed an increase Type I error when the correlation between observations is ignored, these two additional edges are likely to be false positive findings introduced by ignoring the correlation in the data.

**Figure 7:**
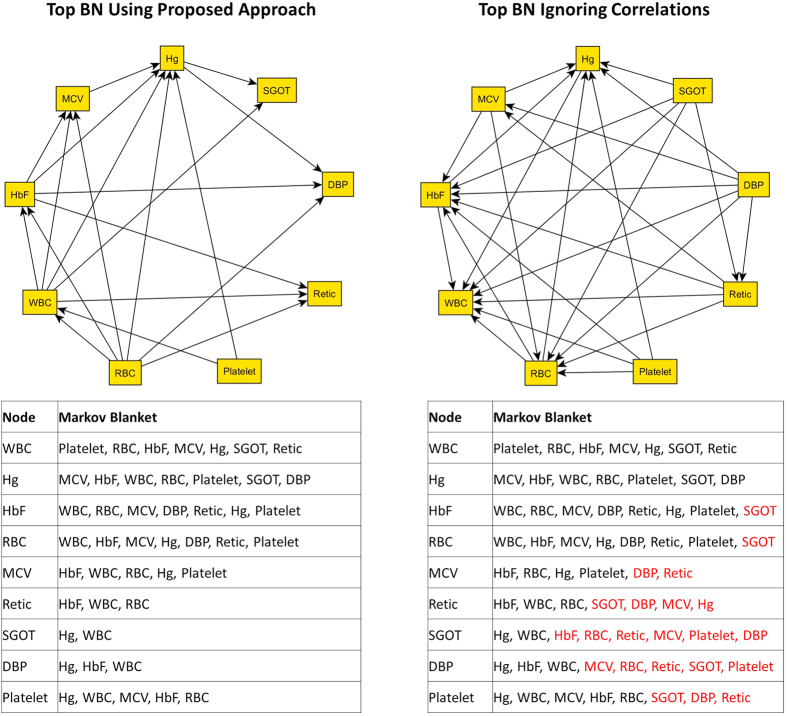
Left Panel: Top BN using the proposed approach and associated Markov Blanket of each node. Right Panel: Top BN built ignoring correlations due to the repeated measurements on the same subjects and associated Markov Blanket of each node. Additional variables in the Markov Blanket as a result of ignoring correlations are colored red. Hg: hemoglobin; SGOT: serum glutamic oxaloacetic transaminase; DBP: diastolic blood pressure; Retic: reticulocyte count; Platelet: platelet count; RBC: red blood cells; WBC: white blood cells; HbF: fetal hemoglobin; MCV: mean corpuscular volume.

In the second example, we used data from 2916 unrelated African-American subjects with sickle cell anemia enrolled in the Cooperative Study of Sickle Cell Disease to model the correlation between several circulating biomarkers of the disease (CSSCD (https://biolincc.nhlbi.nih.gov/studies/csscd/). The data included these 9 biomarkers: fetal hemoglobin, serum glutamic oxaloacetic transaminase, diastolic blood pressure, reticulocyte counts, platelet counts, red blood cell counts, white blood cell counts, hemoglobin, and mean corpuscular volume. Subjects enrolled in the study were followed longitudinally and approximately 3 repeated measures per subject are available, with a total of 8018 measurements available for the current analysis. In order to account for correlations due to repeated measurements on the same subjects, a BN was built by stacking repeated measures and using random effect to describe the correlation between repeated measures of the same study subject. Clinics at which lab measures were taken, age at measurement, and hemoglobin genotypes were considered root nodes of all variables, and all other procedures remained the same as in the previous example. The top BNs and associated MB using the proposed approach and ignoring correlations are illustrated in Fig. 7. Overall, there were 19 edges in the top BN constructed using the proposed approach. When correlations between repeated measures on the same subjects were ignored, there were 28 edges in the BN. These excess edges were reflected as additional variables in the MB of each node, which indicate that virtually all variables are connected to each other. Biologically, the simpler network is more consistent with previous findings that showed strong dependency between hematological parameters, but less dependency of hematological parameters with blood pressure and markers of liver functions (SGOT)^39^. We conjecture that some of these additional edges are likely to be false positives as a result of ignoring apparent correlations between measurements, and this result further bolsters the utility of the proposed approach that can control the false positive error rates for different types of correlation structures.

## Discussion and Conclusions

We presented an approach to learn BNs from correlated data arising from clustered sampling. Our approach uses random effects to model the correlation between observations within the same clusters, and assumes marginal and conditional independence on the random effects to maintain the decomposibility of the likelihood and modularity of the computations. The random effects introduced in the parameterization do not affect the network structure per se, and conceptually they are simply additional random parameters that are useful to model the excess correlation in the data. We evaluated different approximate metrics for model selection in data simulated from a hypothetical family-based study, in which the observations of members within the same family are related with varying degrees of correlation. The simulation study showed the importance of accounting for correlated data to avoid inflation of the false positive error rate, and suggested that in large samples a simple likelihood ratio test based on the integrated maximum likehood may provide a good trade off between false positive and false negative rates. Applications on two real data with different correlation structures showed the potential use of this approach to simultaneously model the associations of genetic and non-genetic factors with a complex trait from a family-based observational study and repeated measures of biomarkers.

Our proposed parameterization can be used for a full Bayesian approach to structural and parameter learnings of BNs with correlated data. However, the selection of BNs from data with many variables is computationally a very challenging problem and therefore we focused the simulation analysis on the evaluation of approximate criteria for model selection. A proper Bayesian approach to model selection of networks learned from correlated data appears to be a very challenging question that needs more work. We limited our analysis to selection of networks with Gaussian data and time-to-event data. In both cases, there is a closed form solution, or good numerical approximation, to the calculation of the integrated likelihood that is used to compute likelihood based criteria such as the likelihood ratio test, *BIC* and *AIC*. However, approximate methods are needed for categorical variables^25^. We also assumed that the vector of random effects followed a normal distribution. Different distributional assumptions on the random effects and mis-specifications of these need to be explored further.

Another popular approach to account for correlations in observations is generalized estimating equation (GEE)^40^. Studies have shown that empirical results on parameter estimation and significance testing are very similar between GEE and random effects models^41^. The advantage of random effect models is that one can carry subject-specific as well as population-average inference, and therefore they provide a more flexible modeling approach for inference.

Our simulations suggest that the likelihood ratio test based on using the integrated likelihood provides a good metric for model selection. The criterion can be interpreted as a crude approximation of the Bayes factor and, compared to *BIC* or *AIC*, it allows users to choose different thersholds for model selection that can trade off sensitivity and specificity. This is an important feature of the criterion, particularly in the analysis of large datasets with several variables.

## Additional Information

**How to cite this article**: Bae, H. *et al.* Learning Bayesian Networks from Correlated Data. *Sci. Rep.* **6**, 25156; doi: 10.1038/srep25156 (2016).

## Supplementary Material

Supplementary Information

## Figures and Tables

**Figure 1:**
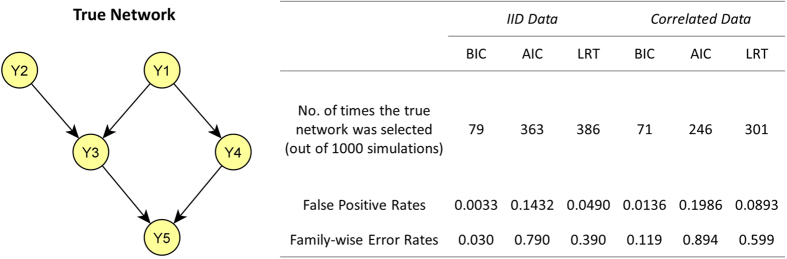
Example of Ignoring Within-Cluster Correlations When Learning BN. 2,000 simulated data sets were generated using the network structure shown on the left and assuming normal distributions for the 5 variables. In 1,000 sets, the observations were IID, and in the remaining 1,000 sets data were generated from 581 independent clusters, with observations correlated within clusters. The table summarizes the number of times the true network was selected in 1,000 simulations with IID observations and 1,000 simulations with correlated data, the false positive rates, and family-wise error rates using three common model selection metrics and a forward search. False positive rates were defined as the number of additional or missing edges over the total number of tests, and family-wise error rates were defined as the probability of one or more errors in the overall search. *BIC*: Bayesian Information Criterion; *AIC*: Akaike Information Criterion; *LRT*: Likelihood Ratio Test at *α* = 0.05.

**Table 1:** False Positive Rates and Family-wise Error Rates of Different Model Selection Metrics For Normally Distributed Data When *h* ^2^ = 0.50.

Score	*n*_*e*_	Number of False Positive Covariates At Each Level					Tot Test	Error Rates	
		1	2	3	4	≥5		FPR	FWER
*BIC*_*M*_	4656	46	0	0	0	0	10405	0.0044	0.045
*BIC*_*J*_	2796	59	1	0	0	0	10530	0.0057	0.058
*BIC*_*Y*_	1768	74	2	0	0	0	10647	0.0071	0.071
*BIC*_*C*_	582	128	7	1	0	0	11135	0.0122	0.120
*AIC*_*M*_	4656	1616	779	270	71	17	23310	0.1181	0.836
*LRT*_*M*_	4656	513	100	13	2	0	14535	0.0432	0.415
*BIC*_*F*_	4656	128	7	0	0	0	11136	0.0121	0.120
*LRT*_*F*_	4656	965	311	79	8	0	18218	0.0748	0.642
*AIC*_*F*_	4656	2316	1381	685	277	110	28479	0.1674	0.931

Levels indicate the hierarchy in the forward search procedure such that Level 1 indicates the search is performed on all 10 covariates, Level 2 indicates that the search is performed on 9 covariates given that at least one false positive covariate was selected in the previous level, and so forth. *BIC*_*M*_: *BIC* based on integrated likelihood and full sample size; *BIC*_*J*_, *BIC*_*Y*_, *BIC*_*C*_: *BIC* with Jones’, Young and conservative effective sample size; *AIC*_*M*_: *AIC* based on integrated likelihood and full sample size; *LRT*_*M*_: likelihood ratio test based on integrated likelihood to account for correlated data; *BIC*_*F*_, *LRT*_*F*_ and *AIC*_*F*_: traditional *BIC*, likelihood ratio test, and *AIC*. *FPR* is the false positive rate defined as number of errors over total number of tests ignoring correlated data; *FWER* is family wise error rate, i.e., probability of one or more errors.

**Table 2:** Power Comparisons of Four Variants of *BIC vs*. Corresponding *LRT*_*M*_ (Normally Distributed Data).

	*α*	Power		
		Strong Effect	Moderate Effect	Weak Effect
*BIC*_*M*_	0.0044	0.572	0.295	0.139
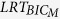	0.0044	0.593	0.314	0.151
*BIC*_*J*_	0.0057	0.608	0.322	0.162
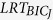	0.0057	0.623	0.340	0.172
*BIC*_*Y*_	0.0071	0.635	0.346	0.178
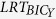	0.0071	0.648	0.362	0.186
*BIC*_*C*_	0.0122	0.708	0.426	0.245
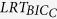	0.0122	0.710	0.429	0.247

Results are based on 1,000 simulated datasets with 3 situations of strong, moderate, and weak covariate effects. *BIC*_*M*_: *BIC* based on integrated likelihood and full sample size; *BIC*_*J*_, *BIC*_*Y*_, *BIC*_*C*_: *BIC* with Jones’, Young and conservative effective sample size; 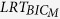, 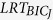, 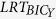, and 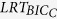: likelihood ratio test based on integrated likelihood using the significance threshold obtained from empirical false positive rates of *BIC*_*M*_, *BIC*_*J*_, *BIC*_*Y*_ and *BIC*_*C*_. For example, since *BIC*_*M*_ has an observed false positive rate of 0.0044, we compared the power of the *BIC*_*M*_ to the power of the *LRT*_*M*_ with significance threshold of 0.0044.

**Table 3:** False Positive Rates and Family-wise Error Rates of Different Model Selection Metrics For Time-to-event Data When *h*^2^ = 0.50.

Score	Number of False Positive Covariates At Each Level					Tot Test	Error Rates	
	1	2	3	4	≥5		FPR	FWER
*BIC*_*M*_	71	1	0	0	0	10638	0.0068	0.070
*AIC*_*M*_	1654	831	327	81	21	23553	0.1237	0.822
*LRT*_*M*_	561	120	14	3	0	14850	0.0470	0.436
*BIC*_*F*_	121	11	0	0	0	11069	0.0119	0.109
*AIC*_*F*_	2057	1180	530	188	58	26572	0.1510	0.884
*LRT*_*F*_	767	226	46	5	0	16604	0.0629	0.543

*BIC*_*M*_: *BIC* based on integrated likelihood and number of events as the sample size; *AIC*_*M*_: *AIC* based on integrated likelihood and full sample size; *LRT*_*M*_: likelihood ratio test based on integrated likelihood to account for correlated data; *BIC*_*F*_, *LRT*_*F*_ and *AIC*_*F*_: traditional *BIC*, likelihood ratio test, and *AIC*. *FPR* is the false positive rate defined as number of errors over total number of tests ignoring correlated data; *FWER* is family wise error rate, i.e., probability of one or more errors.

**Table 4:** Power Comparisons of *BIC*_*M*_ *vs*. Corresponding *LRT*_*M*_ For Time-to-event Data.

		*α*	Power		
			Strong Effect	Moderate Effect	Weak Effect
*h*^2^ = 0.25	*BIC*_*M*_	0.0073	0.961	0.726	0.490
	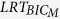	0.0073	0.964	0.741	0.502
*h*^2^ = 0.50	*BIC*_*M*_	0.0068	0.830	0.516	0.315
	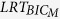	0.0068	0.841	0.522	0.323
*h*^2^ = 0.75	*BIC*_*M*_	0.0078	0.513	0.255	0.144
	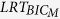	0.0078	0.540	0.285	0.161

Results are based on 1,000 simulated datasets with 3 situations of strong, moderate, and weak covariate effects. *BIC*_*M*_: *BIC* based on integrated likelihood and number of events as the sample size; 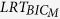: likelihood ratio test based on integrated likelihood using the significance threshold obtained from empirical false positive rates.

**Table 5:** Summary of 23 Genes in the IIS Pathway.

Gene	Chromosome	Number of Tested SNPs
*AKT1*	14	78
*AKT2*	19	142
*AKT3*	1	793
*FOXO1*	13	275
*FOXO3*	6	110
*FOXO6*	1	674
*GHR*	5	506
*IGF1*	12	994
*IGF1R*	15	854
*IKBKB*	8	76
*INS*	11	75
*INSR*	19	859
*IRS1*	2	2105
*IRS2*	13	1637
*PDPK1*	16	9
*PIK3CA*	3	318
*PIK3CB*	3	391
*PIK3CD*	1	172
*PIK3CG*	7	883
*PIK3R1*	5	4179
*PIK3R2*	19	32
*PIK3R3*	1	207
*PIK3R5*	17	295

**Table 6:** Markov Blanket of Each Node in the Top 3 BNs.

Node	MB in *M*1	MB in *M*2	MB in *M*3
FUS	TR, Age.E, Hgb, Sex, rs1009375	TR, Age.E, Hgb, Sex, rs1009375	TR, Age.E, Hgb, Sex, rs1009375
Age.E	BYC, Sex, rs6974881, FUS, TR, Hgb, rs1009375	BYC, Sex, rs6974881, FUS, TR, Hgb, rs1009375	BYC, Sex, rs6974881, FUS, TR, Hgb, rs1009375
DHEA	Hgb, IGF1, BYC, TR	Hgb, IGF1, BYC, TR	Hgb, IGF1, BYC, TR
TR	Hgb, BYC, DHEA, IGF1, FUS, Age.E, Sex, rs1009375	Hgb, BYC, DHEA, IGF1, FUS, Age.E, Sex, rs1009375	Hgb, BYC, DHEA, IGF1, FUS, Age.E, Sex, rs1009375
IGF-1	Hgb, Tr, BYC, DHEA	Hgb, Tr, BYC, DHEA	Hgb, Tr, BYC, DHEA
Hgb	BYC, IGF1, FUS, Tr, DHEA, Age.E, Sex, rs1009375	BYC, IGF1, FUS, Tr, DHEA, Age.E, Sex, rs1009375	BYC, IGF1, FUS, Tr, DHEA, Age.E, Sex, rs1009375

FUS: Follow-up Survival; Age.E: Age at enrollment; DHEA: Dehydroepiandrosterone; TR: Transferrin Receptors; IGF-1: Insulin-like growth factor 1; INS: Insulin; Hgb: Hemoglobin.
