# Supplementary material for: Learning Bayesian Networks from Correlated Data
Source: Sci Rep. 2016 May 5;6:25156. doi: 10.1038/srep25156 (PMC4857179; doi:10.1038/srep25156)
Supplement: Supplementary Information [file srep25156-s1.pdf]

Learning Bayesian Networks from Correlated Data

Harold Bae[1,*], Stefano Monti[2,], Monty Montano[3,], Martin H. Steinberg[2,], Thomas T. Perls[2,], and Paola Sebastiani[4]

[1]Oregon State University, College of Public Health and Human Sciences, Corvallis, 97331, U.S.A.
[2]Boston University, Department of Medicine, Boston, 02118, U.S.A.
[3]Harvard Medical School, Department of Medicine, Boston, 02115, U.S.A.
[4]Boston University, Department of Biostatistics, Boston, 02118, U.S.A.

Supplementary Information

		Errors At Each Level					Tot	Error Rates	
Score	n_e	1	2	3	4	≥5	Test	FPR	FWER
BIC_M	4656	44	0	0	0	0	10396	0.0042	0.044
BIC_J	3346	49	0	0	0	0	10441	0.0047	0.049
BIC_Y	1768	64	0	0	0	0	10576	0.0061	0.064
BIC_C	582	119	2	0	0	0	11042	0.0110	0.114
AIC_M	4656	1598	790	294	77	16	23295	0.1191	0.821
LRT_M	4656	496	96	12	1	0	14336	0.0422	0.397
BIC_F	4656	83	2	0	0	0	10745	0.0079	0.081
LRT_F	4656	727	212	40	3	0	16291	0.0603	0.517
AIC_F	4656	1949	1077	455	152	47	25803	0.1426	0.879

Supplementary Table 1. False Positive Rates and Family-wise Error Rates of Different Model Selection Metrics For Continuous Data When $h^2 = 0.25$. BIC_M: BIC based on integrated likelihood and full sample size; BIC_J, BIC_Y, BIC_C: BIC with Jones', Young and conservative effective sample size; AIC_M: AIC based on integrated likelihood and full sample size; LRT_M: likelihood ratio test based on integrated likelihood to account for correlated data; BIC_F, LRT_F, and AIC_F: traditional BIC, likelihood ratio test, and AIC. FPR is the false positive rate defined as number of errors over total number of tests ignoring correlated data; $FWER$ is family wise error rate, i.e., probability of one or more errors.

Score	n_e	Errors At Each Level 1	2	3	4	≥5	Tot Test	Error Rates FPR	FWER
BIC_M	4656	55	2	0	0	0	10475	0.0054	0.051
BIC_J	2483	64	3	0	0	0	10555	0.0064	0.059
BIC_Y	1768	75	3	0	0	0	10654	0.0073	0.070
BIC_C	582	131	10	0	0	0	11169	0.0126	0.121
AIC_M	4656	1597	758	279	86	18	23135	0.1183	0.822
LRT_M	4656	528	111	16	0	0	14591	0.045	0.415
BIC_F	4656	193	16	0	0	0	11739	0.0178	0.179
LRT_F	4656	1146	447	140	23	1	19781	0.0888	0.702
AIC_F	4656	2540	1582	842	359	159	29816	0.1839	0.936

Supplementary Table 2. False Positive Rates and Family-wise Error Rates of Different Model Selection Metrics For Continuous Data When $h^2 = 0.75$. BIC_M: BIC based on integrated likelihood and full sample size; BIC_J, BIC_Y, BIC_C: BIC with Jones', Young and conservative effective sample size; AIC_M: AIC based on integrated likelihood and full sample size; LRT_M: likelihood ratio test based on integrated likelihood to account for correlated data; BIC_F, LRT_F, and AIC_F: traditional BIC, likelihood ratio test, and AIC. FPR is the false positive rate defined as number of errors over total number of tests ignoring correlated data; $FWER$ is family wise error rate, i.e., probability of one or more errors.

	Power		
	Strong Effect	Moderate Effect	Weak Effect
BIC_M	0.797	0.444	0.273
LRT_{BIC_M}	0.810	0.461	0.287
BIC_J	0.815	0.464	0.290
LRT_{BIC_J}	0.824	0.473	0.297
BIC_Y	0.838	0.510	0.332
LRT_{BIC_Y}	0.835	0.506	0.329
BIC_C	0.877	0.593	0.417
LRT_{BIC_C}	0.875	0.584	0.405

Supplementary Table 3. Power Comparisons of Four Variants of *BIC* *vs*. Corresponding LRT_M For Continuous Data When $h^2 = 0.25$. Results are based on 1,000 simulated datasets with 3 situations of strong, moderate, and weak covariate effects. BIC_M: *BIC* based on integrated likelihood and full sample size; BIC_J, BIC_Y, BIC_C: *BIC* with Jones', Young and conservative effective sample size; LRT_{BIC_M}, LRT_{BIC_J}, LRT_{BIC_Y}, and LRT_{BIC_C}: likelihood ratio test based on integrated likelihood using the significance threshold obtained from empirical false positive rates.

	Power		
	Strong Effect	Moderate Effect	Weak Effect
BIC_M	0.233	0.102	0.049
LRT_{BIC_M}	0.270	0.124	0.067
BIC_J	0.268	0.122	0.067
LRT_{BIC_J}	0.286	0.138	0.072
BIC_Y	0.284	0.138	0.071
LRT_{BIC_Y}	0.304	0.150	0.075
BIC_C	0.356	0.189	0.102
LRT_{BIC_C}	0.374	0.192	0.106

Supplementary Table 4. Power Comparisons of Four Variants of BIC vs. Corresponding LRT_M For Continuous Data When $h^2 = 0.75$. Results are based on 1,000 simulated datasets with 3 situations of strong, moderate, and weak covariate effects. BIC_M: BIC based on integrated likelihood and full sample size; BIC_J, BIC_Y, BIC_C: BIC with Jones', Young and conservative effective sample size; LRT_{BIC_M}, LRT_{BIC_J}, LRT_{BIC_Y}, and LRT_{BIC_C}: likelihood ratio test based on integrated likelihood using the significance threshold obtained from empirical false positive rates.

	Errors At Each Level					Tot	Error Rates	
Score	1	2	3	4	≥5	Test	FPR	FWER
BIC_M	77	1	0	0	0	10683	0.0073	0.075
AIC_M	1661	821	326	91	24	23769	0.1230	0.841
LRT_M	522	114	21	2	0	14646	0.0450	0.413
BIC_F	107	2	0	0	0	10970	0.0099	0.106
AIC_F	1837	956	406	132	39	24851	0.1356	0.864
LRT_F	625	161	25	2	0	15537	0.0523	0.476

Supplementary Table 5. False Positive Rates and Family-wise Error Rates of Different Model Selection Metrics For Time-to-event Data When $h^2 = 0.25$. BIC_M: BIC based on integrated likelihood and number of events as the sample size; AIC_M: AIC based on integrated likelihood and full sample size; LRT_M: likelihood ratio test based on integrated likelihood to account for correlated data; BIC_F, LRT_F, and AIC_F: traditional BIC, likelihood ratio test, and AIC. FPR is the false positive rate defined as number of errors over total number of tests ignoring correlated data; $FWER$ is family wise error rate, i.e., probability of one or more errors.

	Errors At Each Level					Tot	Error Rates	
Score	1	2	3	4	≥5	Test	FPR	FWER
BIC_M	79	4	0	0	0	10707	0.0078	0.075
AIC_M	1712	864	344	106	24	23858	0.1278	0.833
LRT_M	553	124	16	3	0	14910	0.0466	0.434
BIC_F	190	20	1	0	0	11698	0.0180	0.171
AIC_F	2193	1272	597	218	59	27519	0.1577	0.909
LRT_F	906	282	65	14	2	17727	0.0716	0.614

Supplementary Table 6. False Positive Rates and Family-wise Error Rates of Different Model Selection Metrics For Time-to-event Data When $h^2 = 0.75$. BIC_M: BIC based on integrated likelihood and number of events as the sample size; AIC_M: AIC based on integrated likelihood and full sample size; LRT_M: likelihood ratio test based on integrated likelihood to account for correlated data; BIC_F, LRT_F, and AIC_F: traditional BIC, likelihood ratio test, and AIC. FPR is the false positive rate defined as number of errors over total number of tests ignoring correlated data; $FWER$ is family wise error rate, i.e., probability of one or more errors.
